# Supplementary material for: Anti-Cancer and Ototoxicity Characteristics of the Curcuminoids, CLEFMA and EF24, in Combination with Cisplatin
Source: Molecules. 2019 Oct 29;24(21):3889. doi: 10.3390/molecules24213889 (PMC6864451; doi:10.3390/molecules24213889)
Supplement: Supplementary file 1 [file molecules-24-03889-s001.pdf]

molecules

Article

Anti-Cancer and Ototoxicity Characteristics of the Curcuminoids, CLEFMA and EF24, in Combination with Cisplatin

Jerry D. Monroe, Denis Hodzic, Matthew H. Millay, Blaine G. Patty and Michael E. Smith *

Department of Biology, Western Kentucky University, 1906 College Heights Boulevard, #11080, Bowling Green, KY 42101-1080, USA; jerry.monroe@wku.edu (J.D.M.); denis.hodzic021@topper.wku.edu (D.H.); matthew.millay602@topper.wku.edu (M.H.M.); blaine.patty739@topper.wku.edu (B.G.P.)

* Correspondence: michael.smith1@wku.edu

Supplementary Materials

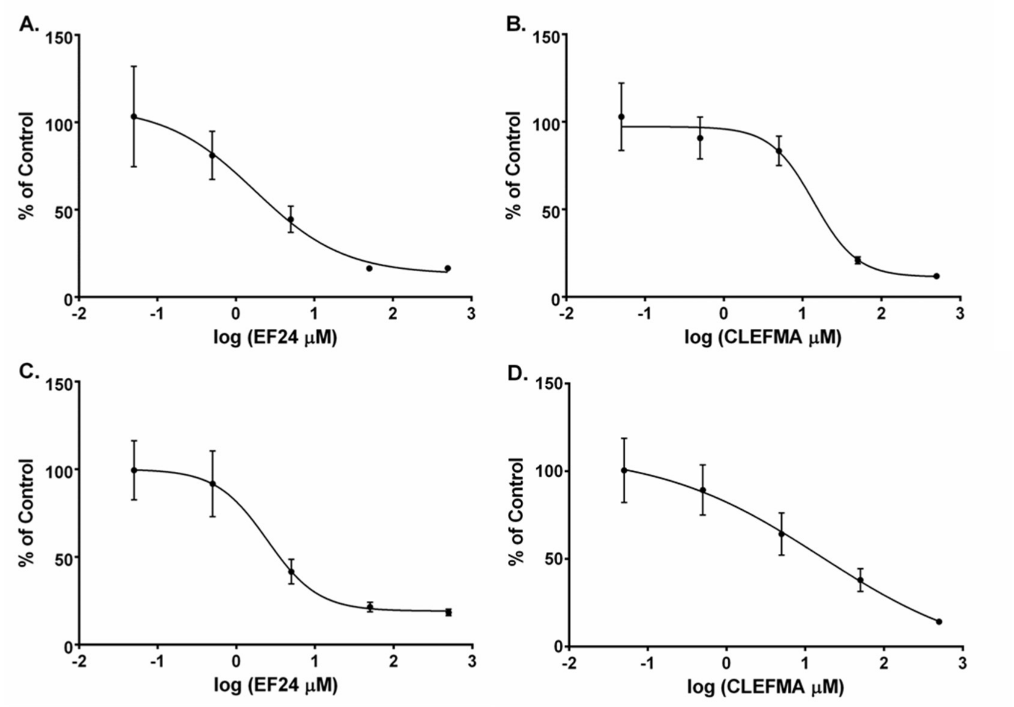

Figure S1. Cellular viability plots for EF24 and CLEFMA 0–24 and 24–48 hour treatments. (**A**) EF24 0–24 hour plot. (**B**) CLEFMA 0–24 hour plot. (**C**) EF24 24–48 hour plot. (**D**) CLEFMA 24–48 hour plot. Plots are shown as a function of MTT assay formazan absorbance as a percent of control versus concentration in μM expressed as a logarithm. The line extending through the data points represents a fit curve derived from the Prism non-linear regression feature. Error bars represent standard deviation.

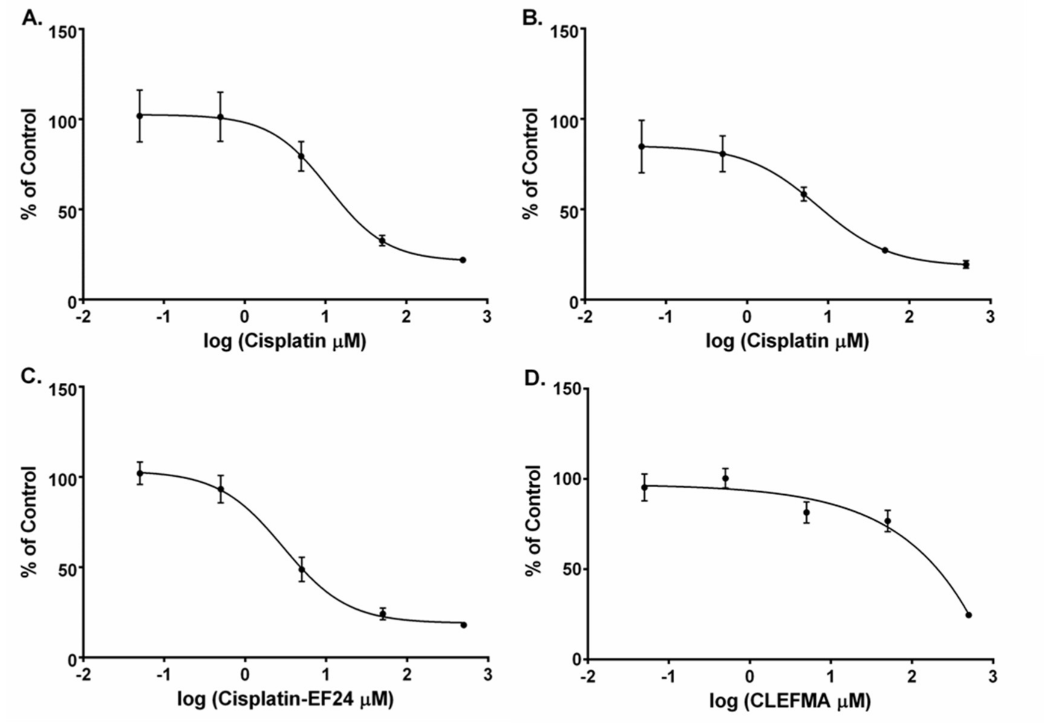

Figure S2. Cellular viability plots for cisplatin 0–24 and 0–48 hour and cisplatin-EF24 and Cisplatin-CLEFMA combination treatments. (**A**) Cisplatin 0–24 plot. (**B**) Cisplatin 0–48 hour plot. (**C**) Cisplatin-EF24 combination plot. (**D**) Cisplatin-CLEFMA combination plot. Plots are shown as a function of MTT assay formazan absorbance as a percent of control versus concentration in μM expressed as a logarithm. The line extending through the data points represents a fit curve derived from the Prism non-linear regression feature. Error bars represent standard deviation.

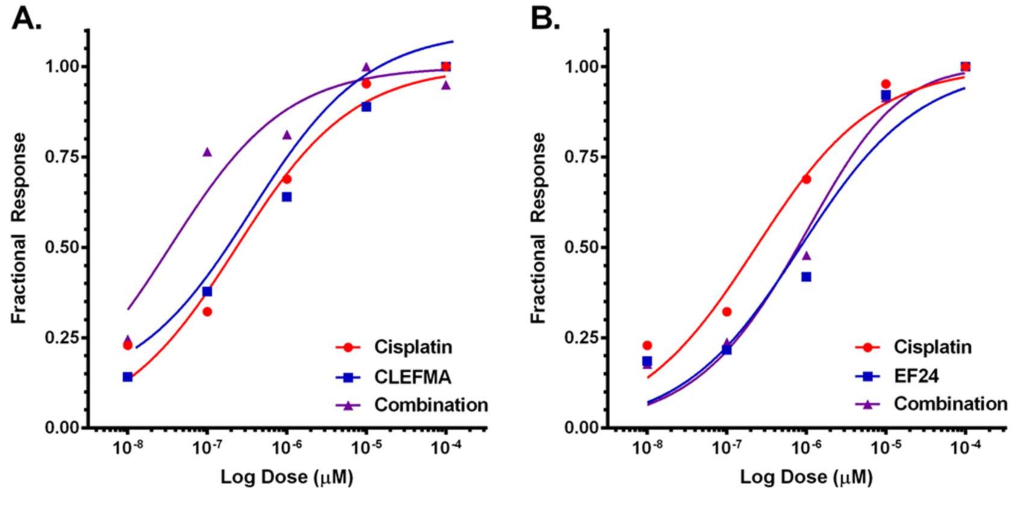

Figure S3. Bliss independence analysis of cisplatin-curcuminoid combination treatments. (**A** and **B**) Key: red line = cisplatin; blue line = CLEFMA or EF24; purple line = combination treatment. (**A**) Cisplatin, CLEFMA and cisplatin-CLEFMA combination dose response curves. (**B**) Cisplatin, EF24 and cisplatin-EF24 combination dose response curves. Plots are shown as a fraction of maximal response versus dose in μM expressed as a logarithm and were made using the Prism nonlinear regression Bliss independence feature set to the 95% confidence limit.

Table S1. Fold change values for auditory evoked potential testing comparisons between experimental treatments and cisplatin or cisplatin vehicle. Hearing thresholds from cisplatin, EF24, CLEFMA, cisplatin-EF24 or cisplatin-CLEFMA combination treated zebrafish were compared with cisplatin or cisplatin vehicle (NaCl) treated fish. Key: "Cisplatin/EF24" = cisplatin followed by EF24, "Cisplatin/CLEFMA" = cisplatin followed by CLEFMA.

	Cisplatin	EF24		CLEFMA		Cisplatin/EF24		Cisplatin/CLEFMA	
**Freq (Hz)**	**Vehicle**	**Vehicle**	**Cisplatin**	**Vehicle**	**Cisplatin**	**Vehicle**	**Cisplatin**	**Vehicle**	**Cisplatin**
100	0.992481	0.986037	1.006536	0.969925	0.977273	0.977444	0.984848	0.947368	0.954545
250	0.998031	0.985377	1.012842	0.968504	0.970414	1.015748	1.017751	0.944882	0.946746
400	0.97379	0.957373	1.017148	0.947581	0.973085	0.967742	0.993789	0.955645	0.981366
600	1.034483	0.982759	1.052632	0.991379	0.958333	1.025862	0.991667	0.978448	0.945833
800	1.082609	1.009938	1.071956	1.004348	0.927711	0.991304	0.915663	0.93913	0.86747
1000	1.087336	1.070493	1.015734	1.048035	0.963855	0.978166	0.899598	0.995633	0.915663
1500	1.08913	1.073292	1.014757	1.056522	0.97006	1.052174	0.966068	1.034783	0.9501
3000	1.028736	1.024631	1.004006	1.034483	1.005587	0.996169	0.968343	0.980843	0.953445

Table S2. Fold change values for auditory evoked potential testing comparisons between experimental treatments and curcuminoid vehicle. Hearing thresholds from cisplatin, EF24, CLEFMA, cisplatin-EF24 or cisplatin-CLEFMA combination-treated zebrafish were compared with curcuminoid vehicle (DMSO) treated fish. Key: "Cisplatin/EF24" = cisplatin followed by EF24, "Cisplatin/CLEFMA" = cisplatin followed by CLEFMA, "Cisplatin/DMSO" = cisplatin followed by DMSO.

	Cisplatin	EF24	CLEFMA	Cisplatin/EF24	Cisplatin/CLEFMA	Cisplatin/DMSO	
**Freq (Hz)**	**DMSO**	**DMSO**	**DMSO**	**DMSO**	**DMSO**	**DMSO**	**Cisplatin**
100	1.015385	1.008791	0.992308	1	0.969230769	0.9692308	0.954545
250	1.038934	1.025761	1.008197	1.057377049	0.983606557	0.9918033	0.954635
400	0.989754	0.973068	0.963115	0.983606557	0.971311475	0.9721311	0.982195
600	1.034483	0.982759	0.991379	1.025862069	0.978448276	0.9827586	0.95
800	1.121622	1.046332	1.040541	1.027027027	0.972972973	0.9774775	0.871486
1000	1.073276	1.05665	1.034483	0.965517241	0.982758621	0.9827586	0.915663
1500	1.035124	1.020071	1.004132	1	0.983471074	0.9793388	0.946108
3000	1.032692	1.028571	1.038462	1	0.984615385	0.9846154	0.953445
